# Benefits of mHealth Co-design for African American and Hispanic Adults: Multi-Method Participatory Research for a Health Information App

**DOI:** 10.2196/26764

**Published:** 2022-03-09

**Authors:** Devlon N Jackson, Neil Sehgal, Cynthia Baur

**Affiliations:** 1 Department of Behavioral and Community Health, Center for Health Literacy, Center for Health Equity School of Public Health University of Maryland College Park, MD United States; 2 Department of Behavioral and Community Health, Center for Health Literacy School of Public Health University of Maryland College Park, MD United States; 3 Department of Health and Policy Management School of Public Health University of Maryland College Park, MD United States

**Keywords:** mHealth app design, health literacy, health disparities, health equity, African Americans, Hispanics, mobile phone

## Abstract

**Background:**

Participatory research methodologies can provide insight into the use of mobile health (mHealth) apps, cultural preferences and needs, and health literacy issues for racial and ethnic groups, such as African Americans and Hispanics who experience health disparities.

**Objective:**

This methodological paper aims to describe a 1-year multi-method participatory research process that directly engaged English-speaking African American and bilingual or Spanish-speaking Hispanic adults in designing a prevention-focused, personalized mHealth, information-seeking smartphone app. We report design team participants’ experiences with the methods to show why our approach is valuable in producing apps that are more aligned with their needs.

**Methods:**

Three design sessions were conducted to inform the iteration of a prevention-focused, personalized mHealth, information-seeking app. The research team led sessions with 2 community member design teams. Design team participants described their goals, motives, and interests regarding prevention information using different approaches, such as collage and card sorting (design session 1), interaction with the app prototype (design session 2), and rating of cultural appropriateness strategies (design session 3).

**Results:**

Each design team had 5 to 6 participants: 2 to 3 male participants and 3 female participants aged between 30 and 76 years. Design team participants shared their likes and dislikes about the sessions and the overall experience of the design sessions. Both African American and Hispanic teams reported positive participation experience. The primary reasons included the opportunity for their views to be heard, collectively working together in the design process, having their apprehension about mHealth reduced, and an opportunity to increase their knowledge of how they could manage their health through mHealth. The feedback from each session informed the following design sessions and a community-engaged process. In addition, the specific findings for each design session informed the design of the app for both communities.

**Conclusions:**

This multi-method participatory research process revealed 4 key lessons learned and recommendations for future research in mHealth app design for African Americans and Hispanics. Lesson 1—community partnerships are key because they provide the *chain of trust* that helps African American and Hispanic participants feel comfortable participating in app research. Lesson 2—community-based participatory research principles continue to yield promising results to engage these populations in mHealth research. Lesson 3—interactive design sessions uncover participants’ needs and development opportunities for mHealth tools. Lesson 4—multiple design sessions with different methods provide an in-depth understanding of participants’ mHealth preferences and needs. Future developers should consider these methods and lessons to ensure health apps in the marketplace contribute to eliminating health disparities and achieving health equity.

## Introduction

### Background

Nationally representative data from the Pew Research Center survey and the Health Information National Trends Survey show that Hispanic and African American adults are more likely than their White counterparts to use smartphones to access health information [1-3]. Hispanic and African American adults use their smartphones to access the internet to search for health information, communicate with health care providers, manage medicines, and use decision support tools, thereby reducing health gaps [1-3]. Despite these new digital options for prevention and management, Hispanic and African American populations are more vulnerable to some of the most common and preventable causes of illness and disease, such as diabetes and heart disease [4,5]. A well-designed, prevention-focused mobile health (mHealth) app that provides credible, culturally appropriate, and easy-to-understand information and recommendations could help better inform these groups, increase information-seeking, and aid decision-making to help prevent or delay chronic diseases. Previous research suggests that intended app users will be more likely to use and benefit from a prevention app if they are informed by their own lived experiences as well as being based on theoretical and methodological approaches that explain health behaviors [6,7]. Such apps can provide examples to mHealth app developers, public health practitioners, and researchers who want to ensure these products help eliminate health disparities, address communication inequities, and achieve health equity which is as follows [8]:

Is achieved when every person has the opportunity to ‘attain his or her full health potential’ and no one is ‘disadvantaged from achieving this potential’ because of social position or other socially determined circumstances.

Limited health literacy may be a major barrier to mHealth app use, specifically among certain populations [9,10]. Healthy People 2030, the United States 10-year health objectives, define health literacy in both personal and organizational terms, and encompass not only comprehension of information but also seeking information and using it for decisions and actions [11]. According to the only nationally representative health literacy study of English-speaking adults in the United States, Hispanics and African Americans had lower average adequate health literacy than their White counterparts [11,12]. It is also important to consider how language is associated with health literacy in relation to the Hispanic population that prefers to speak Spanish or has limited English proficiency for health-related concerns. The National Assessment of Adult Literacy study also revealed that individuals who spoke only Spanish or a language other than English had lower averages of health literacy skills than those whose primary language was English [12]. As the Hispanic population has grown substantially over the past decade, it is imperative that apps are available in Spanish to address their health information needs [13].

Participatory research methods are used in several disciplines, and in mHealth development, these methods have been shown to have positive effects when intended users are part of app development and testing processes [6,14]. This method involves the intended users in the research design and implementation of the intervention, product, or program [15]. Methods to engage intended users in app design and development, such as user-centered design, are known and published in the trade and academic literatures [16-18]. User-centered methods can inform all stages of app development and help refine and update apps as they mature. These methodologies provide insights into the motivations and challenges that users face and can help reveal any special circumstances or requirements that racial and ethnic groups, such as African American and Hispanic adults, may have with mHealth app use when seeking health information to manage their health.

Participatory approaches are imperative to inform and guide research with marginalized communities that have experienced unethical research practices and to engage these communities in research that is not part of their everyday lived experiences. Community-based participatory research (CBPR) has been effective in engaging racial and ethnic groups as well as marginalized communities in the development of mHealth tools. Its success in mHealth is rooted in researchers establishing trust with community partners while collaboratively working on the product development and evaluation processes of the intended product [6,14,19,20]. This is critical when engaging communities who are historically and understandably suspicious of or reluctant to engage in research [21-24].

### Objectives

This methodological paper aims to describe a 1-year multi-method participatory research process that directly engaged English-speaking African American and bilingual or Spanish-speaking Hispanic adults in designing a prevention-focused, personalized mHealth, information-seeking smartphone app. The research reported in this paper was phase 1 of a 4-year process to iteratively refine the field test and revise an app. The study is being conducted by a multidisciplinary team of health literacy, communication, health services, public health, and computer science researchers. The process and app are grounded in CBPR, user-centered design principles, and health literacy techniques. This paper describes (1) the participatory approach, (2) the design session process, (3) participant-reported experiences of the design sessions, and (4) recommendations and lessons learned for future research in mHealth app design for African Americans, Hispanics, and other racial and ethnic groups with a disproportionate burden of health disparities. This paper provides new information about how to combine participatory methods from different intellectual traditions to better discover African American and Hispanic adult health app needs through co-design.

## Methods

### App Design Session Theoretical Underpinnings and Methodological Approach

CBPR principles, user-centered design, and health literacy techniques were our core methods. Understanding the challenges in finding African American and Hispanic adults who might be interested in a research study on an mHealth app, the research team applied several CBPR principles. These principles included (1) building on strengths and resources within the community, (2) promoting a colearning and empowering process that attends to social inequalities, and (3) disseminating findings and knowledge to the user [25]. The research team collaborated with 2 key community partners, a National Institutes of Health–designated Research Center of Excellence in Race, Ethnicity, and Health Disparities Research, and a Hispanic-serving community-based organization. These partners are part of a *chain of trust* in their local Hispanic and African American communities because of the engagement infrastructures they have built over time. By affiliating with these partners, we became part of the chain. Collaboration with the community partners included routine meetings to seek input in the recruitment and retention strategies, content of marketing materials, and input for the development of the design sessions to ensure an equitable participatory research learning process for the participants. In addition, the participant design sessions supported a *co-learning and empowering process that attended to social inequalities* by establishing a bidirectional communicative process between the participants and research team to learn how the communities’ needs can be better served. The research team practiced *disseminating findings and knowledge to the user* by continuously maintaining transparency in the research process and results throughout the study. To put the principles into practice for the app, the team applied user-centered and health literacy techniques.

Not only is a lack of community engagement a weakness in mHealth development but also many tools have been developed without a strong theoretical foundation. Our overall study combined the consumer information process (CIP) model and adult learning theory to inform information-seeking motivation and action [26,27]. The CIP and adult learning theories consider what motivates and engages adults in information-seeking and action. Adult learning theory posits that adults will invest in learning when they perceive a strong *need to know*. The theory’s key ideas about the importance of adults’ previous experience and their developmental stage were applied to inform app design and content. The CIP model’s core concept is *individual processing capacity*, and the model’s core assumption is that individuals are limited and intentional or goal-directed when they seek information and how much information they process [26]. These tenets align with health literacy research and its basic insight that people prefer relevant, easy-to-understand information that does not overwhelm them.

The CIP and adult learning theories and health literacy insights informed the choice of user-centered methods and the design session activities so that the team could develop an understanding of the motivations and interests of the participants in relation to health information–seeking through a prevention-focused mHealth app.

### Background Work on an App Prototype Before the Design Sessions

**Figure 1:**
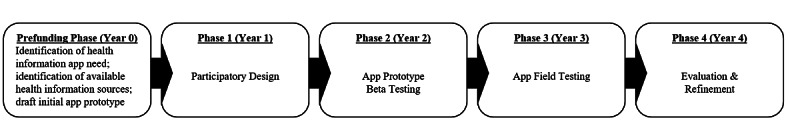
Display of the 4-year smartphone health app research study phases.

Before receiving funding for the 4-year study and implementing the design sessions, the research team conducted a 6-month prefunding phase (year 0) that involved vetting the app concept and developing and programming an early-stage prototype. The core app content is from healthfinder.gov, an award-winning, federal consumer health information website available in English and Spanish that was designed based on health literacy principles [28]. The research team presented this to a community advisory group, the Maryland Community Research Advisory Board (MD-CRAB), to validate the app concept, receive general feedback on the prototype, and determine what amendments should be made before applying for research funding to support the phases shown in Figure 1. The MD-CRAB is a research advisory group established by the Maryland Center for Health Equity (M-CHE) at the University of Maryland School of Public Health. The MD-CRAB includes 22 members, most from the local African American and Hispanic communities, and they provide community insight and guidance to strengthen research and ensure the results of research benefit vulnerable populations, especially in African American and Hispanic populations.

The African American and Hispanic MD-CRAB members reported that they believed people such as themselves might be willing to use an app that provided (1) information specific to their medical concern, (2) an array of health information, (3) bilingual options, and (4) culturally tailored health information. We used this feedback to iterate the prototype and plan the research funding proposal. Once the study was funded in August 2018, we planned and began implementing phase 1 design sessions.

### Design Session Participant Recruitment

Between September 2018 and December 2018, the research team worked with 2 community partner organizations in Prince George’s and Montgomery Counties, Maryland, United States, to recruit English-speaking African American and bilingual or Spanish-speaking Hispanic adults. The organizations advised us on culturally tailored recruitment fliers. M-CHE led the African American recruitment, and the M-CHE Director asked the local barbers and stylists in the research community network in Prince George’s County, Maryland, United States, to share the fliers. Requests to share the fliers with church members were made to local African American churches. An African American research team member presented the project at local church meetings to recruit participants. The research team also tried recruiting at local, free, well-attended health services events by many African American and Hispanic community members. Community health workers at Community Health and Empowerment through Education and Research (CHEER), a nonprofit service provider for Hispanic residents in the Takoma Park, Maryland neighborhood, distributed fliers and asked their clients to volunteer for the study. Interested African American and Hispanic community members called the project telephone number and left a message for a bilingual research assistant who called back and screened callers in English or Spanish to see if they met the study criteria. The goal was to recruit 1 group of African American adults and 1 group of bilingual or Spanish-speaking Hispanic adults who would commit to attending multiple design sessions over several months.

We screened participants based on race and ethnicity, age, sex, education, smartphone access, information-seeking habits, and willingness to participate in multiple sessions. Participants had to self-identify as African American or Hispanic or Latino and aged ≥18 years. We aimed for an equal distribution of self-identified male and female participants. As healthfinder.gov, the app’s core content, is designed to be useful for adults with low health literacy, we were interested in working with community members with low health literacy and low health information–seeking habits. We screened participants to identify those who had no more than 2 years of community or technical college, had a smartphone or were willing to learn to use one, and answered questions that indicated they were a low to medium health information seeker and had low health literacy based on questions from the Health Information National Trends Survey [29]. Those who met the recruitment criteria were scheduled for the initial African American or Hispanic design sessions.

### Design Session Planning

The research team wrote the moderators’ guides in English that the bilingual research assistants translated into Spanish. The guides provided informed consent, followed by open-ended questions and tasks specific to the design sessions. The purpose of the sessions was to confirm the basic structure of the prototype app and solicit intended user ideas and feedback for a revised app with additional content, features, and functions. We asked the same questions of the African American adults (in English) and Hispanic adults (in Spanish). A sample moderator’s guide in English is available for review in the Multimedia Appendix 1. We scheduled the African American Study Director to lead the African American sessions and either a CHEER community health worker or a Hispanic bilingual research assistant to lead the Hispanic sessions in Spanish. We audio recorded each session and scheduled at least one bilingual or English-speaking research assistant to take notes during each session, depending on the session (English or Spanish). The design sessions were held on Saturdays at a local church for African American participants and evenings at the CHEER offices, a Hispanic-serving community-based organization, to make them convenient for participants based on their availability and preferences. Building trust and comfort between ourselves and participants was why we chose to hold the 3 sessions with the same 2 groups of participants. Participants were offered a gift card for US $25 for each session attended and an additional remuneration of US $25 if they completed all 3 sessions.

### Ethics Approval

Each design session received approval from the University of Maryland Institutional Review Board (approval numbers 1292902-1, 1388156-1, and 1430335-1), and all project team members completed the Collaborative Institutional Training Initiative training for human subjects research before engaging with participants.

### Design Session Procedures

**Figure 2:**
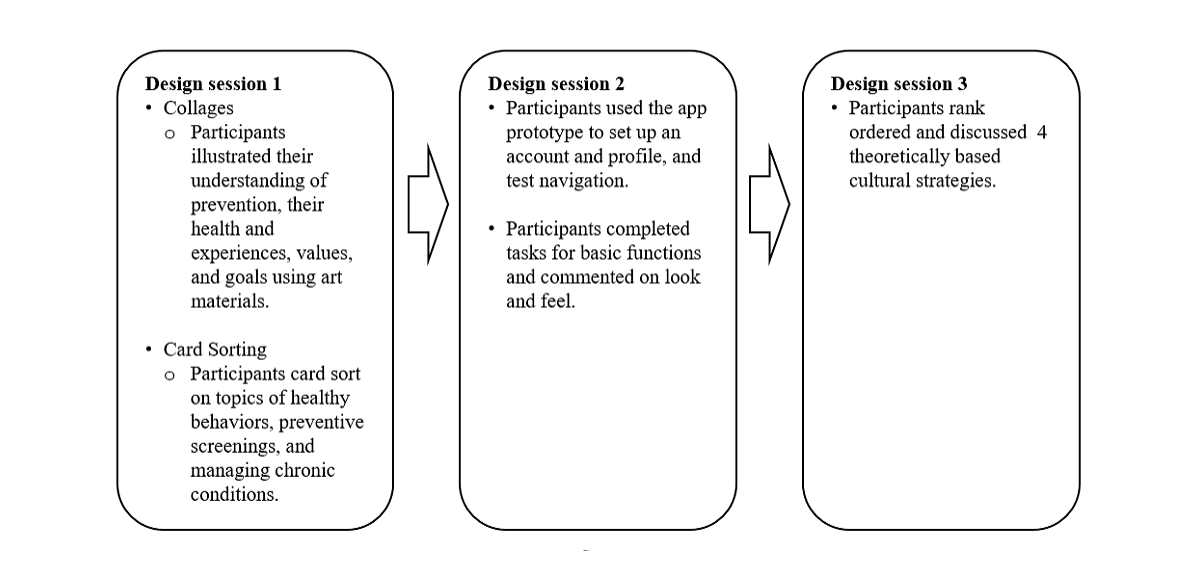
Design session activities.

User-center design methods engage the intended users of a product or service at different stages of design and testing processes to ensure maximum usability [30,31]. Our community partner organizations told us that although many community members have smartphones, they might not know much about how to download and use apps. In addition, we did not know how comfortable participants would be sharing information about their health experiences, beliefs, and technology use. As we were also applying CBPR and health literacy principles in how we engaged with participants, we chose methods to allow participants to draw on what was familiar to them and become comfortable talking about health topics and smartphone apps. We used hands-on activities and plain language in our study materials and design discussions to help participants fully understand and share the design process. To minimize any literacy challenges, we read aloud written materials, such as informed consent materials, written directions, or information on screens. During the 3 design sessions, we wanted participants to think broadly about their health and describe their goals, motives, and interests regarding prevention information using different approaches: (1) collage and card sorting (design session 1), (2) interaction with the app prototype (design session 2), and (3) rating of cultural appropriateness strategies (design session 3; Figure 2). In addition, participants could share their likes and dislikes about the sessions and the overall experience of the design sessions. We followed the same process as our separate African American and Hispanic groups for each design session.

### Design Session 1 Process

The goal of design session 1 was to learn participants’ meanings and interpretations of health, prevention, and management, and the collage and card sorting activities involved participants creatively expressing their ideas, thoughts, interests, and desires about health generally and their health specifically. Collages are a *make tool* for people to create artifacts and express unspoken feelings and emotional states [32]. Participants were provided with tools such as poster board, colored pencils, markers, and printed images of people and activities that allowed them to create collages to explain what prevention meant to them, describe their present health, express their health values, and what they desired for their health in the future. Each participant had 30 minutes to create their own collage, and then the group spent 30 minutes on a *walking tour* so that each participant could tell the story of their collage to the group. The 1-hour card sorting activity allowed participants to have a tangible task to discuss prevention topics, which can be abstract for many people, and took advantage of in-depth group discussion to learn about participants’ thoughts about the cards presented to them [33]. The card sorting activity involved each participant individually receiving a card stack on a group of health topics. The topic groups were healthy behaviors such as physical activity and healthy eating; preventive screenings and vaccinations, such as blood pressure checks or annual flu shots; and managing chronic conditions, such as chronic back pain or diabetes. We asked participants if they had questions about the terms on the cards and to talk aloud for 10 to 15 minutes about what the topics meant to them.

At the end of the discussion, we asked them to choose 2 to 3 cards that were most relevant to them. We repeated this process for round 2 with preventive screenings and vaccinations. For round 3, we asked them to look at 5 chronic disease cards and tell us the first thing they thought of. At the end of the session, we collected the collages and stacks of the sorted cards for analysis. The research assistants summarized the recordings and their session notes and organized responses by discussion questions in spreadsheets. The research team members who attended the session, which included the principal investigator (PI) and the study director, debriefed, examined the collages for similarities and differences, and reviewed participants’ comments by question. The PI and study director discussed the collages and participants’ comments and recommended app changes to the full research team, who collectively decided what to revise before design session 2.

### Design Session 2 Process

The goal of design session 2 was to learn how participants navigated the app prototype, and the session involved participants interacting with the revised prototype app and providing input on the interface, functions, and overall look and feel. A key step in user-centered design is to provide prototypes to a small number of intended users to try real-world tasks and scenarios [30]. Our participant groups included people with vision or literacy challenges; so, at the session sites we set up full-size computer monitors and connected a smartphone with the app to display the app screens for easier readability. The participant, research team facilitator, and research assistant sat around the monitor, and then the participant held the smartphone to complete the tasks while being observed. We read aloud the directions and information. We video recorded each session, and the research assistant took notes. Participants had 4 tasks to complete: (1) review the introductory content of the app that describes the app purpose, (2) perform a nonpersonalized health information search in the app, (3) perform a basic personalized health information search in the app entering data about their age, sex, and pregnancy status, and (4) create a personalized profile with demographic and health information data. We asked them to talk aloud while they did the tasks and explain what they were doing and thinking. The research assistants summarized the recordings and notes of participants and questions in spreadsheets. The PI and the study director, who were present during the testing, reviewed the responses and recommended app changes to the full research team for discussion and implementation decisions.

### Design Session 3 Process

Knowing and including culturally appropriate references is a key tenant of effective health communication practice, which can also be applied to digital health tools [34,35]. The goal of design session 3 was to learn design team participants’ cultural preferences and needs in relation to health, and we explored culturally appropriate strategies reported in the literature that are necessary for relevant and useful health information and messages [34,36]. On the basis of comments and suggestions from design sessions 1 and 2 as well as the literature on culturally tailored strategies, the research team developed a packet of activities that asked participants to evaluate the different strategies: (1) cultural appropriateness appearance, (2) evidence of health issues specific to African Americans and Hispanics, (3) specific language and linguistic content, and (4) sociocultural values and characteristics. For the appearance evaluation task, for example, the packet included possible images for the app, and for evidence, examples of health statistics relevant to either African American or Hispanic adults. The research team discussed each strategy with participants. This session also involved participants viewing and ranking their interest in web content and tools, such as a health services locator, nutrition planner, medication information source, and an air quality tracker, that the research team was considering for the final app. These activities provided further understanding of what the participants would like to integrate in the app to increase and motivate engagement.

The research assistants summarized the audio recordings and their written notes on the spreadsheets. The PI and the study director summarized the discussions for the full research team, and the team reviewed participants’ app content addition preferences. The research team convened the design groups in a separate final community member report-back meeting. The research team shared the revised app with the design session participants so they could see how their input manifested itself in the app. This was a *report-back* and a user validation session, not data collection, to close the loop with our community co-designers. The authors took notes but did not audio record the final session.

### Process for How Design Session Data Informed the App

Each session was audio or video recorded, and sometimes both depending on the type of session, and transcribed and the Spanish-speaking sessions were translated into English by bilingual research assistants on the team. Data collected from each session were entered into spreadsheets and categorized based on the design session discussion guide questions. The input from each design session informed the app development and was organized according to the (1) changes to the existing prototype to make it more attractive, easier to use, and relevant; (2) missing features that participants felt would motivate their app use and continued engagement; (3) suggestions on how to present and explain the importance of preventing illness and disease; and (4) suggestions on how to explain the personalization features, such as personal and family health history information and goal-setting. The analysis of the data from each design session is not included in this paper, as the intent of this methodological paper is to propose and discuss the multi-method co-design process we used. This paper provides the feedback from the design session team participants’ on their experiences participating in the overall design process. Participant feedback was provided informally during each design session.

## Results

### Design Session Recruitment and Attendance

The M-CHE recruitment efforts at one local African American church resulted in 6 English-speaking African American adults, and the CHEER outreach produced 5 bilingual or Spanish-speaking Hispanic adults participating in the sessions (Table 1). The racial, ethnic, and sex distributions were 3 African American women, 3 African American men, 3 Hispanic women, and 2 Hispanic men. Participants’ were aged between 30 and 76 years. Three design sessions and a report-back in English for African American participants and 3 design sessions and a report-back in Spanish for Hispanic participants occurred between December 2018 and May 2019. The groups were conducted separately to allow each group to communicate in their preferred language. Each design session lasted 2 to 3 hours. Although we held the sessions at the local church or CHEER office, participant attendance varied by session, sometimes resulting in 5 to 6 participants attending.

**Table 1:** Design session participant demographics (n=11).

Demographics			African American participants (n=6)		Hispanic participants (n=5)
Age (years), range			30-76		35-76
**Sex, n (%)**					
	Male	3 (50)		2 (40)	
	Female	3 (50)		3 (60)	
Total number of participants (design session 1), n (%)			6 (100)		5 (100)
Total number of participants (design session 2), n (%)			5 (84)		5 (100)
Total number of participants (design session 3), n (%)			5 (84)		5 (100)

### Design Session Participant Experiences

#### Overview

Both African American and Hispanic design team members reported a positive participation experience. The primary reasons for their experiences included (1) the opportunity for their views to be heard, (2) collectively working together in the design process, (3) having their apprehension about mHealth reduced, and (4) an opportunity to increase their knowledge of how they could manage their health through mHealth. This feedback informed us about how we approached each design session and how we engaged the participants. In addition, the specific findings for each design session (not reported in this methodology paper) informed the design of the app for both communities.

#### Reason 1: Opportunity for Their Views to Be Heard

Both African American and Hispanic participants said that having their *perspectives* heard was important. Hispanic participants reported the following:

I like that you take our views and perspectives into account as elders and facilitate access to cell phone research.

I appreciate very much that you are taking all of us into account.

#### Reason 2: Collectively Working Together in the Design Process

African American participants reported the following: “I agree with everyone but I think the gathering, us networking, being able to share our experiences, being able to talk about ourselves openly and not being judgmental about what somebody else is going through.”

This quote also reflected another reason for participants’ positive experiences, as it reflects their appreciation of working together in the design process. Participants felt valued and respected, resulting in comfortably sharing their honest opinions with one another and the research team about what they thought of the app. Another participant reported the following: “In regards to the app I think that it is actually a good tool that we are part of and that you guys are trying to put out there for people to have more knowledge and have more control of their health.”

In addition, an African American participant inquired how the research team established a relationship with the community to conduct this research, and we explained how the community partnerships connected us to the participants. The participant responded by saying the following:

I think that this is a good thing because we really don’t have enough of that [research] and with so much going on...we need to do research...

#### Reason 3: Having Their Apprehension About mHealth Reduced

The third reason for the participants’ positive experience with using the app was the reduced apprehension about mHealth. An African American participant reported the following:

You guys explaining everything to me. My vision is bad and I suffer sometimes when I’m looking at things. But you guys made it easy for me by assisting me. I’ve kind of understood and I’m not lost. At first I was concerned because I thought, ‘Oh gosh, I’m going to sit here and be lost. I’m not going to be able to see this...etc.’ But you guys made sure that I’m comfortable, I’m not intimidated...

This particular participant reported apprehension while using mHealth technologies but wanted to be involved in the study to learn how to use the technology and overcome their fear. Their comment also provided insight into the app function that needed to be adjusted based on physiological abilities.

#### Reason 4: An Opportunity to Increase Their Knowledge of How They Could Manage Their Health Through mHealth

Finally, the other positive experience participants reported was an opportunity to increase their knowledge of how they could manage their health through mHealth. A Hispanic participant stated the following:

I like what they (the research team) are doing (the session) at the University of Maryland for the Hispanic community. There are many times that they (Hispanic community) don’t find or don’t know that a stomach ache or something simple can become something chronic. I like it [the app] a lot.

African American participants reported the following:

That’s what I got out of it. The app enables you to take care of yourself.

## Discussion

### Principal Findings

#### Overview

Our study design aimed to create a positive app development experience for a small group of English-speaking African American and Spanish-speaking Hispanic adults and generate rich data to inform an app development project. The mHealth literature reports the importance of community participation and user-centered design, and participants’ responses to the research methods reported in this paper confirm these strategies. However, this study went further than others in applying health literacy principles at every step of the mHealth design process—from recruitment of participants to selection of app content, feature design, and the report-back process. Our study provides 4 key lessons and recommendations in mHealth Design for racial and ethnic groups with health disparities [37,38].

#### Lesson 1: Community Partnerships Are Key in Engaging Racial and Ethnic Groups With a Disproportionate Burden of Health Disparities in mHealth App Development Research

The research team in collaboration with the community partner organizations was able to successfully recruit and retain the necessary number of participants over a 6-month design session series. We tapped into the *chain of trust* that our community partners had with the intended app users. The research team remained connected with the community partner organizations to discuss strategies to engage Hispanics and African Americans in the research process and app design. For example, when the research team and community partners were initially designing recruitment materials (ie, fliers and recruitment screening tools), both community partners stated that one standard flier for both groups would not be effective. On the basis of their experience with the local community members, the African American flier required a message stating how their community would benefit from the technology if they participated. The community partner stated that African Americans are constantly bombarded with negative statistics and information about their community, and they want to hear information framed as gains rather than losses (eg, what we will gain if we participate). The Hispanic community partner advised us to frame their recruitment message as both the gains and losses of healthier lifestyle adoption (eg, our community can gain new information and will lose out if we do not participate). Similar input informed design session moderator guides and session materials. Discussions with community partners also revealed similarities in how African American and Hispanic participants would prefer to engage in design sessions as it relates to the type of materials used, topics discussed, and technology preferences. The constant feedback and support from the community partners helped create an engaging design session environment that produced the positive and transparent participant experiences reported. We were able to apply cultural tailoring not only to the app but also in the development of the design sessions. Routine interaction and feedback with the community partners assisted us in navigating and avoiding potential pitfalls that might have occurred had we not established the collaboration.

#### Lesson 2: Application of CBPR Principles Continues to Yield Promising Results When Engaging Racial and Ethnic Groups With a Disproportionate Burden of Health Disparities in mHealth Research

Owing to the equitable and colearning environment that CBPR brings to the research environment, people are more likely to see value in participating in the research process because they see how their input is valued while also gaining additional information and resources. Several participants stated they appreciated that they had the opportunity to share their input about a product intended for them. A large body of literature demonstrates the benefits of applying some or all of the CBPR principles when engaging community members in research [39-41]. CBPR is also being applied specifically in mHealth app development and encouraged by other mHealth and public health researchers and developers [6,14,42,43].

#### Lesson 3: Interactive Design Sessions Allow Individuals to Uncover Their Needs and Opportunities for the mHealth Tool Being Developed

Several of the design session participants reported that participating in the sessions increased their awareness of health issues and the need for an mHealth information-seeking app. When presented with the prototype app for review, several reported not knowing that this information was or could be available to them and also stated how helpful it would be for themselves and others they know. Adult learning theory posits that adults will invest in learning when they perceive a strong *need to know*, and an mHealth information-seeking app can be the prompt. The team’s design approach provides a tangible method that allows participants to uncover their needs and opportunities for the mHealth tool. It allows participants to manipulate something and discover something new in the process.

#### Lesson 4: Sustained Design Sessions Using Multiple Approaches Can Provide an In-depth Understanding in mHealth Preferences and Needs in Appearance, Function, and Content

Participants had the opportunity to provide their views about health and its relationship to information-seeking through various methods. These types of approaches aid developers and help them avoid unnecessary and potentially biased errors in the development process [6]. The use of design sessions that built on each other (eg, collaging, card sorting, user–app interaction, and cultural appropriateness strategies) allowed the research team and participants to learn about each other and develop a deeper understanding of what motivates and maintains engagement in mHealth apps among health disparity populations.

### Strengths and Limitations

Two of our study’s strengths focus on health literacy issues for smartphone app design and language access for people who prefer health information in languages other than English. Excluding higher education and information-seeking participants extended recruitment because many people who expressed interest did not meet these 2 criteria. However, we felt it was important to work with participants with limited health literacy and design an app for their needs that would likely work well for others with higher health literacy [28]. Working with English- and Spanish-speaking groups in parallel allowed us to see cultural and language similarities and differences in how groups might vary in app use, although we did not set the goal of conducting an explicit comparative study.

The extended engagement over 4 sessions with the same group of African American and Hispanic adults is also a strength of our study. We were able to use multiple methods to collect participant insights on different app design issues, and our participants informed our decisions at different development stages and experienced app development along with the research team. Furthermore, our app developers attended many design sessions, and they could visualize and hear participants in their own words, which kept them grounded in our participants’ lived experiences.

Having participants participate in real-time app development also had its limitations. They could not experience the app in its fully functional form as we were learning and prototyping based on their input. The research team continued to iterate the app for several months after the design sessions concluded. One key feature of the app, a personalization algorithm, was added during the final development phase. We described the algorithm and personalization of the participants, but they could not try it in real time.

Although our design groups had a typical number of participants (5 to 6) for these types of user-centered design studies, a limitation is the small number of participants, as they cannot represent the needs, experiences, and perspectives of all African American and Hispanic smartphone users. However, the multiple design sessions with the same participants performing concrete tasks allowed them to provide detailed information not available through other methods. As other researchers note, user-centered methods have high information yields and rich data [37].

### Conclusions

Our study contributes to the small and growing literature on the involvement of marginalized groups in mHealth design and evaluation. Although health literacy and language access issues are cited as barriers to some groups’ engagement with mHealth, our project shows how careful attention to these issues can be incorporated into standard user-centered methods and CBPR principles. Participants’ comments about how much they appreciated the sessions and the chance to engage with mHealth tools on their own terms validates our approach.

To reduce cultural bias errors, sustain engagement, and provide culturally relevant mHealth information apps to African American and Hispanic adults who continue to be affected disproportionately by preventable chronic conditions, developers should consider a multifaceted participatory research process that includes user-centered design and health literacy approaches. Because of social factors that increase their vulnerability to chronic diseases, it is imperative that these communities have access to digital tools that truly address their health information needs. Although health information–seeking apps are not a common or popular type of app, they have significant potential to serve as a tool that can support individuals’ health care management. The comments from our participants indicate that these communities would greatly benefit from an mHealth information-seeking tool that is personalized to their health information needs. Future developers should consider more integrative app development approaches to ensure that the apps on the market contribute to eliminating health disparities and achieving health equity. It is the responsibility of those who are developing mHealth apps to ensure that studies are inclusive of the communities they intend to serve.

## Appendix

Multimedia Appendix 1 Sample moderator guide in English for a design session (session 1 of 3 sessions).

## Abbreviations

CBPR: Community-Based Participatory Research
CHEER: Community Health and Empowerment through Education and Research
CIP: consumer information process
M-CHE: Maryland Center for Health Equity
MD-CRAB: Maryland Community Research Advisory Board
PI: principal investigator
