# Supplementary material for: Benefits of mHealth Co-design for African American and Hispanic Adults: Multi-Method Participatory Research for a Health Information App
Source: JMIR Form Res. 2022 Mar 9;6(3):e26764. doi: 10.2196/26764 (PMC8943540; doi:10.2196/26764)
Supplement: Multimedia Appendix 1 [file formative_v6i3e26764_app1.docx]

Moderator’s Guide for Collage and Card Sort Activity with African American and Latino Adults: Input to *HealthyMe/MiSalud* Smartphone Application

Total estimated session length: 3 hours

One session will be in English and one session in Spanish. Final version of the guide will be in English and Spanish.

Reference: <http://www.designkit.org/methods/25> and <http://www.designkit.org/methods/24>

**Project Introduction (5 minutes)**

Hello, my name is Dr. Cynthia Baur. I am the project director, and this is my colleague, Dr. Devlon Jackson. (*Note: In the Spanish-speaking group, a bilingual graduate student will lead the session, and Drs. Baur and Jackson will be present*.) We’re from the University of Maryland School of Public Health, and we’re part of a team developing a smartphone application or app for adults like yourselves to find out how to get and stay healthy and set health goals. The working title of the app is *HealthyMe*. We appreciate very much that you’ve agreed to be part of our team while we work on a test version of the app. Today/tonight is the first of three sessions we’ll do together during the next 6 months.

Tonight’s session has two activities that will help us understand how you think about your health. The first activity is telling your story about your health through a picture that you create, and the second activity is about sorting words and ideas about health. We hope you have fun with these activities. There aren’t any right or wrong answers, and everyone’s opinions and ideas are equally valuable to the project.

Before we begin the activities, we will go through the informed consent process with you as a group. Informed consent means that we have provided you understandable information about the study, we have answered any questions you have, and you agree to participate in the study. (*Pass out informed consent forms*.). I will read the form aloud and pause for questions as we go. You can stop me if you don’t understand something or have a question. You can still change your mind and don’t have to participate if we don’t address your concerns. Ok, let’s begin. (*Read consent form, answer questions, ask participants to sign, release anyone who doesn’t want to sign, collect signed forms*.)

**Participant Introductions (15 minutes)**

Let’s begin with introductions. Please say your first name, what interested or motivated you to be part of this group, and one thing in your life you’re proud of. (*Introductions*) Thank you.

**Activity 1: Collage about health (1 hour)**

Our first activity involves using the materials we’ve provided – paper, pencils, markers, pictures, glue, tape, and scissors – to create a story about your health through words and images. On the top, please fill in your first name, gender, and age. On the left side, we’ve marked a section for you to show how you feel your health is most days, and on the right side how you would like your health to be in the future. Across the bottom, please show what is most important to you about your health. You can use words, draw pictures, cut and paste from the pictures anything you’d like to do to express how you feel about your health. You can begin right away or take a few minutes to think about your ideas. We have about 30 minutes for this activity. At the end of 30 minutes, we’ll take a walking tour and visit everyone’s picture. We’ll ask each of you to take about 5 minutes to describe your picture. Then we’ll take a 10 minute bathroom and refreshment break and move to activity #2. What questions do you have? Ok, let’s begin.

**Break (10 minutes)**

**Activity 2: Card sorting (1 hour)**

Our second activity uses cards that have words on them. We’ll give each of you a stack of cards and ask you to talk about and sort them in groups. What questions do you have? Ok, let’s begin.

Sort #1: Here are some cards with words about physical activity, food, and preventing diseases such as diabetes and heart disease. I will read the cards as I put them on the table and then we’ll talk about what they mean to you. Cards will have words about physical activity, healthy eating behaviors, and cancer, heart, and diabetes prevention.

*Discuss* for 10-15 minutes what participants say about the cards.

Card words:

exercise; physical activity; working out; get moving; going to the gym; walking briskly; jogging; cycling; gardening; swimming; healthy eating; nutrition; healthy eating patterns; fill half your plate with fruits and veggies; calories; manage weight; overweight; obesity

Thank you for those comments. Now I’d like you to put the 2-3 cards that are most relevant or interesting to you in a pile. Thank you, I’ll collect these and we’ll go to the next set of cards.

Sort #2: Here are some cards with words on them. We’ll give each of you a stack of cards and ask you to talk about and sort them in groups. What questions do you have? Ok, let’s begin.

Cards will have words about preventive health actions, including getting a shot, mammogram, and colonoscopy; blood pressure check; images of a person looking sad with words “depression screening” and a person looking at a bottle of alcohol with words “talk with doctor about alcohol use.”

*Discuss* for 10-15 minutes what participants say about the cards.

Card words: All these words are related to talking to a doctor or other provider about the topic, getting a shot, or taking a test to see if you have a disease or health condition:

mental health screening; oral health screening; immunizations; genetic screening; mammogram; colonoscopy; blood pressure; cholesterol; tobacco use screening; alcohol use screening; sexually transmitted infections screening; violence screening; injury screening; prevent cancer; prevent diabetes; prevent heart disease; prevent strokes; prevent obesity; getting access to care

Thank you for those comments. Now I’d like you to put the 2-3 cards that are most relevant or interesting to you in a pile. Thank you, I’ll collect these and we’ll go to the next set of cards.

Sort #3: Here are some cards with words about different diseases and health conditions. . Please tell us the first thing you think of. We don’t want you to think too long.

Show participants each card one at a time:

dealing with chronic back pain; staying healthy with a disability; living with heart disease; living with diabetes; living with arthritis

**Debrief, participant questions, and preview sessions 2-4 (15 minutes)**

Thank you for your time and participation tonight. You’ve done great work that is important to how we will develop the smartphone app. We’ll use the ideas to create the next version of the app and bring a working version and smartphones for you to use to our next session. The next 2 sessions will be about using the app and giving us feedback about how it works and what we should revise or add.

As a reminder, the next sessions are scheduled for XXX. You will receive reminder texts about session #2 one week and one day before.

How did you find tonight’s process? What did you like? What didn’t you like? Recommendations for using this process with other people like yourselves? (*Open discussion*)

Thank you again, and good night. We will pass out your gift cards as you pack up. Please drive safely.
